# Supplementary material for: Towards future directions in data-integrative supervised prediction of human aging-related genes
Source: Bioinform Adv. 2022 Nov 2;2(1):vbac081. doi: 10.1093/bioadv/vbac081 (PMC9710570; doi:10.1093/bioadv/vbac081)
Supplement: vbac081_Supplementary_Data [file vbac081_supplementary_data.pdf]

Supplementary Material for: Towards future directions in data-integrative supervised prediction of human aging-related genes

October 25, 2022

S1 Supplementary Sections

S1.1 Data: Downloading GTEx raw data

The GTEx GE data was curated using RNA-seq technology, which can be downloaded from GTEx Portal (`https://gtexportal.org/`). We use data resulting from the GTEx Analysis V8, which is the latest version that has RNA-seq GE data available. In particular, among all V8 data files in GTEx Portal, we use the RNA-seq gene read counts data called "GTEx_Analysis_2017-06-05_v8_RNASeQCv1.1.9_gene_reads.gct.gz" as our resource to obtain GE data. The age group information about each sample in the gene read counts data can be found from "GTEx_Analysis_v8_Annotations_SubjectPhenotypesDS.txt" in the GTEx Portal.

S1.2 Data: aging-related GO terms

The structure of GO can be viewed as a "tree-like" graph, where nodes are GO terms and edges are "loosely hierarchical" relationships between GO terms [Ashburner et al., 2000, Gene Ontology Consortium, 2021]. It is referred to as "loosely hierarchical" [Ashburner et al., 2000] because a "child" GO term could have more than one "parent" GO term. "Child" GO terms are more specialized compared to their "parent" GO terms in the graph. The root GO term of the aging process is GO:0007568[1]. We obtain all aging-related GO terms that are "child" GO terms of the aging GO term (GO:0007568) tree. Moreover, because the p-values of enrichment tests are typically greater than 0.046 if a set of size is smaller than 3 [Cao and Zhang, 2014], it is not appropriate to question the significance of enrichment for a small sample size [Zheng and Wang, 2008]. Therefore, we only consider aging-related GO terms that annotate at least three genes in ground truth labeled genes. As such, we obtain 13 such aging-related GO terms. We list the size and the number of genes that are annotated by each GO term and are labeled in Supplementary Table S5. Note that gene-GO term annotations have different evidence codes indicating their curation in accordance. That is, those annotations with experimental evidence codes (EXP, IDA, IPI, IMP, IGI, IEP) are the most confident ones. So, we also further consider aging-related GO terms and all their experimentally validated annotations (counts can be found in the 4-th column of Supplementary Table S5).

We test enrichment in aging-related GO terms for the four gene groups. There are typically two options to select aging-related GO terms. The first option is that one can consider all aging-related GO terms, regardless of the evidence codes about how the gene-GO term annotations are obtained. As such, we obtain 13 aging-related GO terms that each GO term annotates at least three genes in ground truth data (Supplementary Table S5). In this case, on average each GO term annotates 13 genes in ground truth data. Because gene-GO term annotations with experimental evidence codes are more trustworthy (confident), the second option is that one can only consider aging-related GO terms whose annotations are experimentally validated. As such, we obtain eight aging-related GO terms that each GO term annotates at least three genes in ground truth data (Supplementary Table S5). In this case, on average each GO term annotates only 5.25 genes in ground truth data. If we consider the second option, we don't have enough data and statistical power to claim the significance of our enrichment tests [Newaz et al., 2020, Zheng and Wang, 2008]. Therefore, we only focus on the first option in aging-related GO term enrichment analysis.

[1] `http://www.informatics.jax.org/vocab/gene_ontology/GO:0007568`

S1.3 Predictive models

Recall that a predictive model is a feature-classifier combination. For the classifier, we use logistic regression for all predictive models. Intuitively, logistic regression is a binary classification algorithm that relies on a logistic function to assign the probability of an entity being classified as the class of interest among the two binary classes. The reasons why we do this are stated below. In particular, first, in our previous series of studies with respect to supervised prediction of aging-related genes [Li and Milenković, 2019, 2021, Li et al., 2021], we tested nine prominent classifiers. Of all nine prominent classifiers, logistic regression consistently performed well, i.e., consistently being selected as the classifier component of the best predictive model for a given (weighted or unweighted, dynamic or static) aging-specific subnetwork. Second, logistic regression is well known for its robustness in various supervised classification tasks [Yu et al., 2011].

For the network feature, among six types of features proposed in our previous study [Li et al., 2021], we select two types of features that consistently performed well, i.e., consistently being selected as the feature component of the best predictive model for a given weighted dynamic aging-specific subnetwork. In particular, for a given node, *(1)* the Pearson correlation of weight distribution among its up to two-hop neighbors, and *(2)* the weight distribution of edges among its up to two-hop neighbors, are the two types of features considered in this study. Within each feature type, we consider four network neighborhood types. That is, for a node v in a given subnetwork, we count the frequencies of each weight in the subnetwork among edges of the following four types: first, edges that are directly connected to the node v; second, edges that are not directly connected to the node v, but are connecting any pairs of v's direct neighbors (i.e., v's one-hop neighbors); third, edges that are connecting any pairs of v's direct neighbors (one-hop neighbors) and v's two-hop neighbors; fourth, edges that are connecting any pairs of v's two-hop neighbors.

Given our considered weighted dynamic aging-specific subnetwork with w unique edge weights across its n differential snapshots, we first compute the corresponding four types of the neighborhood for a given node v in a snapshot. The two feature types are obtained as follows.

- For feature type-*(1)*, for each of the $n*(n-1)/2$ snapshot pairs, we first calculate the Pearson correlation of edge weights of node v across the considered two snapshots in the given pair. By this step, each node in the subnetwork has a $n*(n-1)/2$-dimensional feature vector. Then, we repeat the process for each of the four neighborhood types, which results in four type-*(1)* feature vectors for each node in the subnetwork. In addition to the four type-*(1)* features, we consider the fifth feature by concatenating the four type-*(1)* features, resulting in a $4*n*(n-1)/2$. In total, we consider five type-*(1)* feature vectors for each node in the subnetwork. We refer to this feature type as cor-$\langle i \rangle$, where $i = 1, 2, 3, 4, 5$ represents each of the four neighborhood types and the concatenation of the four neighborhood types.
- For feature type-*(2)*, given a snapshot, we count the frequency of each of the w weights among v's given node neighborhood type. By this step, each node has a feature vector of w. Then, we repeat the process for each of the n snapshots, and concatenate feature vectors across all snapshots. As such, we obtain $w \times n$-dimensional feature vector for node v for each of the four neighborhood types. Consequently, we obtain four types-*(2)* feature vectors for each node in the subnetwork. Unlike type (1), we do not concatenate the four type-*(2)* features due to the computational infeasibility. We refer this feature type to as raw-$\langle i \rangle$, where $i = 1, 2, 3, 4$ represents each of the four neighborhood types.

In total, we consider nine features, i.e., feature types (each feature is a vector), each coupled with the logistic regression to form a predictive model. In other words, we consider nine predictive models for each considered weighted dynamic aging-specific subnetwork.

S1.4 Evaluation framework

S1.4.1 Evaluation in terms of 5-fold cross-validation

Given our defined 277 aging- and 4,282 non-aging-related gene labels from GenAge, we test nine predictive models for each of the four considered weighted dynamic aging-specific subnetworks for cross-validation proposed in our previous study [Li et al., 2021]. The evaluation process can be illustrated as the following steps.

1. We randomly and equally split the 277 aging- and 4,282 non-aging-related genes into five subsets, respectively. Each fold (a combination of a subset of aging-related genes and a subset of non-aging-related genes) is considered as testing data at a time, and the remaining four folds combined are

considered as training data. To ensure fair comparison among all four networks, we intentionally force the randomly split five folds of aging- and non-aging-related genes the same across all four subnetworks. As such, we perform 5-fold cross-validation for each predictive model.

2. Recall that to give each subnetwork the best case of advantage, other than varying the feature component of a predictive model, we also perform hyperparameter training for logistic regression. That is, we further perform 5-fold cross-validation to find the best hyperparameter for our classifier − logistic regression during the training process. In particular, given a fold of training data, we randomly split it into five folds, of which one fold is treated as tuning-testing data and the remaining four folds are treated as tuning-training data. We select 10 regularization strength hyperparameter values of logistic regression in the log space between −8 and 8. The ten values are selected using python 'NumPy' package, i.e., $numpy.logspace(-8, 8, num = 10, base = 2.0)$. We select the best hyperparameter that yields the highest average AUPR across the five-fold tuning-testing data.

3. After selecting the best hyperparameter from the tuning process for a given fold of training data, we use the selected hyperparameter to retrain the logistic regression using the entire fold of training data, and predict whether a gene in the corresponding fold of testing data is aging-related or not. Specifically, we rank the list of genes in the testing data according to the predicted probability of how likely a gene is aging-related, i.e., from high to low. Then we predict k genes on the top of the output as aging-related. We vary k from 1 to $\lceil(277 + 4282)/5\rceil = 912$ with an increment of 1.

4. We repeat the above two steps five times, and calculate their average AUPR, average precision, average recall, average F-score over the five folds. In particular, for each k, we count the corresponding true positives (i.e., predicted as aging-related that are GenAge-based aging-related genes), false positives (i.e., predicted as aging-related but are not GenAge-based aging-related genes), and false negatives (i.e., not predicted as aging-related but are GenAge-based aging-related genes). With these numbers calculated for each k across five folds, we then calculate the average AUPR, average precision, average recall, and average F-score. Precision = true positives / (true positives + false positives); recall = true positives / (true positives + false negatives); F-score = 2 × precision × recall / (precision + recall). The AUPR is the area under the precision and recall curve. Finally, we sort prediction accuracy scores in decreasing order for each k using average AUPR as primary sort index, average F-score as secondary sort index, then average precision and recall as the third and fourth sort indices, respectively; and we select the first k in the sorted list, referred to as $k1$ that yields best prediction accuracy. As such, the given predictive model predicts $k1$ genes as aging-related, with the four prediction accuracy scores obtained at threshold $k1$.

5. Given nine considered predictive models for a subnetwork, we select the best predictive model that yields the highest average AUPR. If tied on average AUPR, then select the one that yields the highest average F-score.

S1.4.2 Statistical test of prediction accuracy

To test whether a subnetwork is statistically significantly better than another subnetwork or better than expected by chance, we use the paired Wilcoxon signed-rank test [Wilcoxon, 1992]. We do this for each pair of two subnetworks and each pair of a subnetwork plus random approach. Because these statistical tests reside in the same background, we apply false discovery rate correction [Benjamini and Hochberg, 1995]. Just as typically done in literature, we set our significance level as 0.05. Note that, to get prediction accuracy that is expected by chance, we mimic the 5-fold cross-validation, by randomly selecting k genes in the testing data and predicting them as aging-related. We repeat such process 30 times and account for the four prediction accuracy measures over $30 \times 5 = 150$ random runs.

S1.4.3 Validating the complementarity of two gene sets

To measure whether two sets of genes (X, Y) are complementary to each other, we use the Jaccard index (i.e., $J(X,Y) = \frac{X \cap Y}{X \cup Y}$) to measure their overlap. To test whether the overlap is random or statistically meaningful, we use hypergeometric test [Rivals et al., 2007]. Similarly, we set the significance level as 0.05, and use false discovery rate correction to adjust p-values. We use these measures to *(1)* validate whether two subnetworks are predicting complementary aging-related genes to each other; *(2)* test whether our four gene

groups (i.e., 'Predicted-Aging', 'Predicted-NonAging', 'NotPredicted-Aging', 'NotPredicted-NonAging') are enriched in aging-related pathways and GO terms.

S1.5 Mapping feature vectors on the 2-dimensional vector space

To map the high-dimensional feature vectors into 2-dimensional (2D) vector space for visualization purposes, we use two prominent dimensionality reduction methods, i.e., principal component analysis (PCA) and t-distributed stochastic neighbor embedding (tSNE) [Maaten and Hinton, 2008].

PCA is a *linear* dimensionality reduction method, by projecting data points to principal components to obtain lower-dimensional vector space, to preserve the data's variation as much as possible in the top few components. To map a feature vector into 2-D dimensional space, one can simply take the first two principal components (most important two principal components). tSNE is a *nonlinear* dimensionality reduction method that reduces high-dimensional feature vector into a 2D vector space. It is commonly used for visualization and dimensionality reduction purposes. tSNE is sensitive to perplexity values, which indicates the number of nearest neighborhoods considered to balance the attention between local and global perspectives of the data. We consider six values within the range of $[5, 50]$ suggested by tSNE's original publication [Maaten and Hinton, 2008], i.e., 5, 13, 21, 30, 40, and 50.

For each subnetwork, we visualize the feature that corresponds to the best selected predictive model of the subnetwork using both PCA and six tSNE versions. Then, we present the one with a clear separation between our four gene groups, i.e., 'Predicted-Aging', 'Predicted-NonAging', 'NotPredicted-Aging', 'NotPredicted-NonAging'.

S1.6 GenAge selection criteria

GenAge provides information about why a gene was selected for inclusion in the database. Here, we dive deep aiming to understand whether there is a specific group that our subnetworks are more or less successful in predicting. We gather all such information for our 277 aging-related genes and analyze how our true positive predictions are distributed in the different GenAge gene selection criteria groups, see Supplementary Table S6. Moreover, we also perform hypergeometric tests on whether a subnetwork's coverage of a selection criterion is statistically significant. Our results show that only GTEx-HPRD and Predicted-Aging's coverage of downstream selection criteria are statistically significant (i.e., adjusted p-values are 0.035 and 0.013, respectively). Other than that, our predictions are distributed roughly evenly across different selection criteria groups along with the group sizes (i.e., adjusted p-values > 0.05).

S2 Supplementary Tables

Supplementary Table S1: The prediction accuracy in terms of average AUPR, F-score, precision, and recall of the nine predictive models for Berchtold-HPRD. The number in parenthesis is the corresponding standard deviation for a given subnetwork and a given prediction accuracy measure. The highest accuracy scores and the selected "best" predictive models are in bold. Note that we name the predictive models based on their feature components (see Supplementary Section S1.3 for details about each feature). The "# of predictions" in the table is the number of true positives and false positives (i.e., novel predictions). The corresponding Figure presentation is Supplementary Fig. S1.

Predictive model	# of predictions	Average AUPR	Average F-score	Average precision	Average Recall
Cor-1	265	0.38 (0.07)	0.39 (0.07)	0.40 (0.08)	0.38 (0.07)
Cor-2	215	0.45 (0.10)	0.46 (0.08)	0.52 (0.09)	0.40 (0.07)
Cor-3	215	0.48 (0.10)	0.46 (0.10)	0.52 (0.11)	0.40 (0.09)
Cor-4	205	0.48 (0.10)	0.47 (0.07)	0.55 (0.09)	0.41 (0.06)
Cor-5	180	**0.49 (0.10)**	0.48 (0.09)	0.61 (0.11)	0.40 (0.07)
Raw-1	190	**0.49 (0.09)**	0.47 (0.08)	0.58 (0.10)	0.40 (0.07)
Raw-2	215	**0.49 (0.11)**	0.50 (0.08)	0.57 (0.09)	**0.44 (0.07)**
**Raw-3**	175	**0.49 (0.08)**	**0.52 (0.09)**	**0.67 (0.12)**	0.42 (0.07)
Raw-4	185	0.43 (0.10)	0.48 (0.07)	0.61 (0.09)	0.40 (0.06)

Supplementary Table S2: The prediction accuracy in terms of average AUPR, F-score, precision, and recall of the nine predictive models for Berchtold-BioGRID. The number in parenthesis is the corresponding standard deviation for a given subnetwork and a given prediction accuracy measure. The highest accuracy scores and the selected "best" predictive models are in bold. Note that we name the predictive models based on their feature components (see Supplementary Section S1.3 for details about each feature). The "# of predictions" in the table is the number of true positives and false positives (i.e., novel predictions). The corresponding Figure presentation is Supplementary Fig. S2.

Predictive model	# of predictions	Average AUPR	Average F-score	Average precision	Average Recall
Cor-1	210	0.20 (0.03)	0.27 (0.02)	0.31 (0.03)	0.23 (0.02)
**Cor-2**	220	**0.44 (0.07)**	**0.47 (0.06)**	**0.53 (0.07)**	**0.42 (0.05)**
Cor-3	270	0.37 (0.06)	0.42 (0.05)	0.43 (0.05)	**0.42 (0.05)**
Cor-4	265	0.24 (0.04)	0.33 (0.05)	0.34 (0.05)	0.32 (0.05)
Cor-5	235	0.43 (0.07)	0.46 (0.06)	0.50 (0.07)	**0.42 (0.05)**
Raw-1	245	0.30 (0.03)	0.38 (0.04)	0.40 (0.04)	0.35 (0.04)
Raw-2	160	0.31 (0.04)	0.39 (0.05)	**0.53 (0.07)**	0.31 (0.04)
Raw-3	160	0.14 (0.03)	0.22 (0.04)	0.29 (0.06)	0.17 (0.03)
Raw-4	170	0.32 (0.04)	0.37 (0.03)	0.48 (0.04)	0.30 (0.02)

Supplementary Table S3: The prediction accuracy in terms of average AUPR, F-score, precision, and recall of the nine predictive models for GTEx-HPRD. The number in parenthesis is the corresponding standard deviation for a given subnetwork and a given prediction accuracy measure. The highest accuracy scores and the selected "best" predictive models are in bold. Note that we name the predictive models based on their feature components (see Supplementary Section S1.3 for details about each feature). The "# of predictions" in the table is the number of true positives and false positives (i.e., novel predictions). The corresponding Figure presentation is Supplementary Fig. S3.

Predictive model	# of predictions	Average AUPR	Average F-score	Average precision	Average Recall
Cor-1	230	0.30 (0.07)	0.32 (0.05)	0.35 (0.06)	0.29 (0.05)
Cor-2	205	0.41 (0.10)	0.41 (0.09)	0.48 (0.10)	0.35 (0.07)
Cor-3	225	0.45 (0.11)	0.46 (0.11)	0.51 (0.13)	0.42 (0.10)
Cor-4	170	0.48 (0.11)	0.47 (0.09)	**0.61 (0.11)**	0.38 (0.07)
Cor-5	230	0.48 (0.10)	0.49 (0.08)	0.53 (0.09)	0.44 (0.07)
Raw-1	245	0.43 (0.10)	0.44 (0.08)	0.47 (0.08)	0.41 (0.07)
Raw-2	220	0.46 (0.10)	0.47 (0.08)	0.53 (0.10)	0.42 (0.08)
**Raw-3**	225	**0.50 (0.09)**	**0.51 (0.08)**	0.57 (0.09)	**0.46 (0.07)**
Raw-4	195	0.37 (0.09)	0.44 (0.08)	0.53 (0.10)	0.37 (0.07)

Supplementary Table S4: The prediction accuracy in terms of average AUPR, F-score, precision, and recall of the nine predictive models for GTex-BioGRID. The number in parenthesis is the corresponding standard deviation for a given subnetwork and a given prediction accuracy measure. The highest accuracy scores and the selected "best" predictive models are in bold. Note that we name the predictive models based on their feature components (see Supplementary Section S1.3 for details about each feature). The "# of predictions" in the table is the number of true positives and false positives (i.e., novel predictions). The corresponding Figure presentation is Supplementary Fig. S4.

Predictive model	# of predictions	Average AUPR	Average F-score	Average precision	Average Recall
Cor-1	220	0.14 (0.02)	0.17 (0.04)	0.19 (0.05)	0.15 (0.04)
Cor-2	280	0.23 (0.04)	0.28 (0.04)	0.28 (0.04)	0.28 (0.04)
Cor-3	230	0.23 (0.03)	0.31 (0.06)	0.34 (0.06)	0.28 (0.05)
Cor-4	285	0.18 (0.03)	0.26 (0.05)	0.26 (0.05)	0.26 (0.05)
Cor-5	240	0.21 (0.04)	0.28 (0.05)	0.30 (0.05)	0.26 (0.04)
Raw-1	280	0.24 (0.04)	0.32 (0.03)	0.32 (0.03)	**0.32 (0.03)**
Raw-2	180	0.24 (0.01)	0.34 (0.02)	0.43 (0.03)	0.28 (0.02)
Raw-3	155	0.13 (0.03)	0.18 (0.03)	0.25 (0.05)	0.14 (0.03)
**Raw-4**	140	**0.31 (0.08)**	**0.35 (0.08)**	**0.52 (0.11)**	0.26 (0.06)

Supplementary Table S5: Statistics about 13 considered aging-related GO terms. The second column shows the number of genes (i.e.,size) annotated by a given GO term. The third column shows the number of genes annotated by a given GO term that are also present in ground truth. The fourth column shows the number of genes annotated by a given GO term that are experimentally validated and are present in ground truth. The GO terms are sorted by their IDs.

GO term	Size	# of annotated genes that are present in ground truth	# of annotated genes that are experimentally validated and are present in ground truth
Aging<br>GO:0007568	141	71	4
Cell aging<br>GO:0007569	25	13	4
Determination of adult lifespan<br>GO:0008340	18	6	1
Multicellular organism aging<br>GO:0010259	25	12	1
Negative regulation of cell aging<br>GO:0090344	8	5	3
Cellular senescence<br>GO:0090398	44	18	9
Replicative senescence<br>GO:0090399	16	13	8
Stress-induced premature senescence<br>GO:0090400	4	3	2
Oncogene-induced cell senescence<br>GO:0090402	4	3	2
Oxidative stress-induced premature senescence<br>GO:0090403	4	3	2
Modulation of age-related behavioral decline<br>GO:0090647	21	5	3
Negative regulation of cellular senescence<br>GO:2000773	20	12	8
Positive regulation of cellular senescence<br>GO:2000774	10	3	3

Group	GenAge (277)	Berchtold-HPRD (117)	Berchtold-BioGRID (116)	GTEx-HPRD (128)	GTEx-BioGRID (73)	Predicted-Aging (167)
functional	74	37 (50%)	34 (45.9%)	37 (50%)	18 (24.3%)	47 (63.5%)
putative	69	16 (23.2%)	15 (21.7%)	21 (30.4%)	9 (13%)	28 (40.6%)
downstream	56	31 (55.4%)	29 (51.8%)	36 (64.3%)	22 (39.3%)	45 (80.4%)
mammal	53	16 (30.2%)	20 (37.7%)	15 (28.3%)	13 (24.5%)	26 (49.1%)
upstream	34	18 (52.9%)	15 (44.1%)	19 (55.9%)	13 (38.2%)	20 (58.8%)
model	27	12 (44.4%)	15 (55.6%)	13 (48.1%)	9 (33.3%)	19 (70.4%)
cell	23	14 (60.9%)	15 (65.2%)	15 (65.2%)	8 (34.8%)	20 (87.0%)
human_link	5	1 (20%)	0 (0%)	1 (20%)	1 (20%)	2 (40%)
human	3	1 (33.3%)	1 (33.3%)	1 (33.3%)	1 (33.3%)	2 (66.7%)
Total	344	146	144	158	94	209

Supplementary Table S6: The number of genes in each GenAge selection criterion group. We include all considered 277 GenAge aging-related genes, the true positive predictions from each of the four subnetworks, and the union of true positive predictions of all four subnetworks (i.e., the column named "Predicted-Aging"). From second column onward, the number following a column name in parenthesis represents the total number of unique genes. Note that a gene can be included in GenAge due to multiple selection criteria. This is why the total sum of the number of genes (i.e., the last row) across all GenAge selection criteria groups for a given set is bigger than the set size represented in the first row. For example, there are 277 unique genes in GenAge, and some of the genes belong to multiple GenAge selection criteria groups. Thus, the SUM(74, 69, ..., 3) = 344, which is greater than 277.

S3 Supplementary Figures

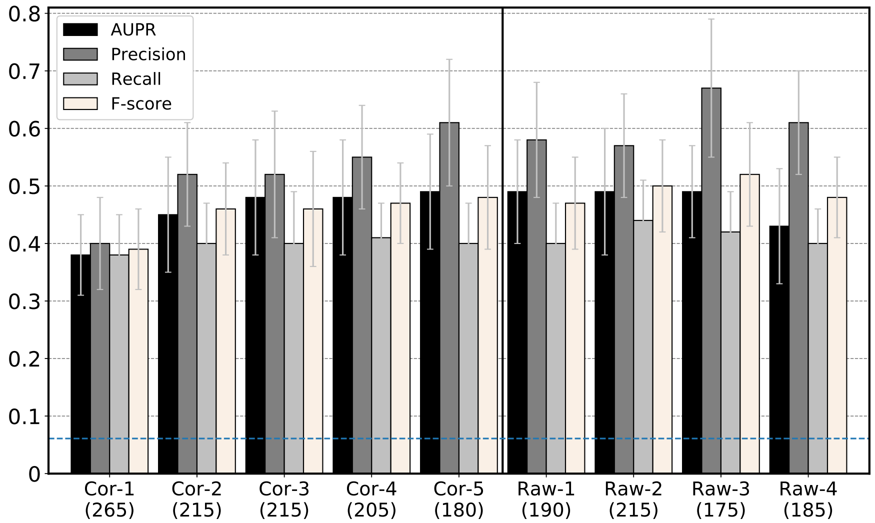

Supplementary Fig. S1: The prediction accuracy in terms of AUPR, precision, recall, and F-score of the nine predictive models for Berchtold-HPRD. The nine predictive models are named after their feature component. The number below the name of each predictive model represents the number of genes that are predicted as aging-related. The blue dashed line indicates the prediction accuracy scores expected by chance, i.e., the fraction of all genes in the ground truth data that are labeled as aging-related.

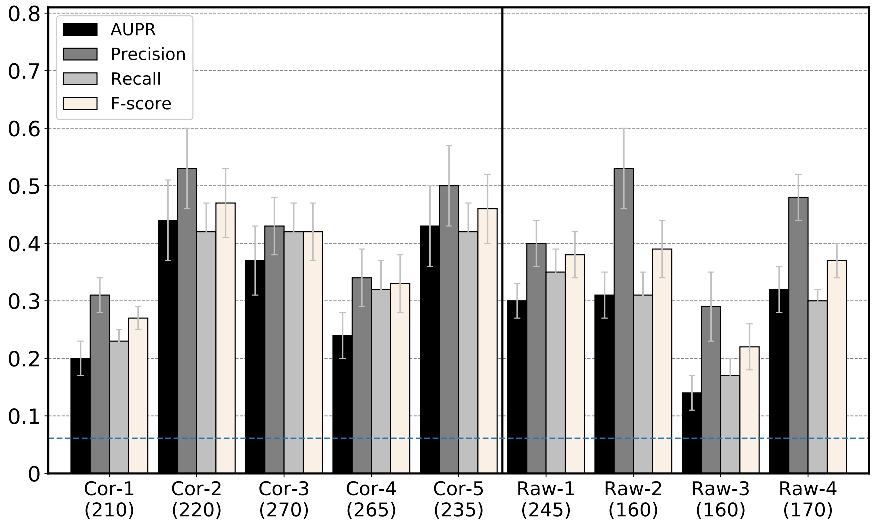

Supplementary Fig. S2: The prediction accuracy in terms of AUPR, precision, recall, and F-score of the nine predictive models for Berchtold-BioGRID. The nine predictive models are named after their feature component. The number below the name of each predictive model represents the number of genes that are predicted as aging-related. The blue dashed line indicates the prediction accuracy scores expected by chance, i.e., the fraction of all genes in the ground truth data that are labeled as aging-related.

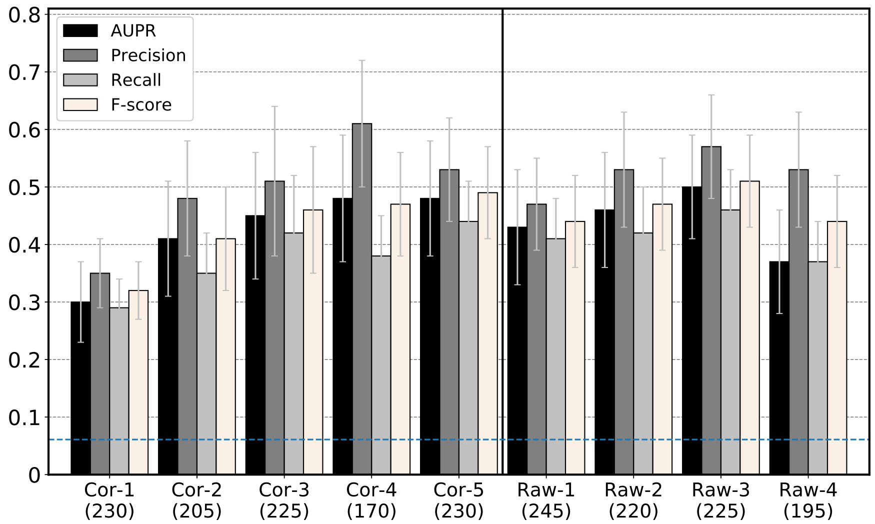

Supplementary Fig. S3: The prediction accuracy in terms of AUPR, precision, recall, and F-score of the nine predictive models for GTEx-HPRD. The nine predictive models are named after their feature component. The number below the name of each predictive model represents the number of genes that are predicted as aging-related. The blue dashed line indicates the prediction accuracy scores expected by chance, i.e., the fraction of all genes in the ground truth data that are labeled as aging-related.

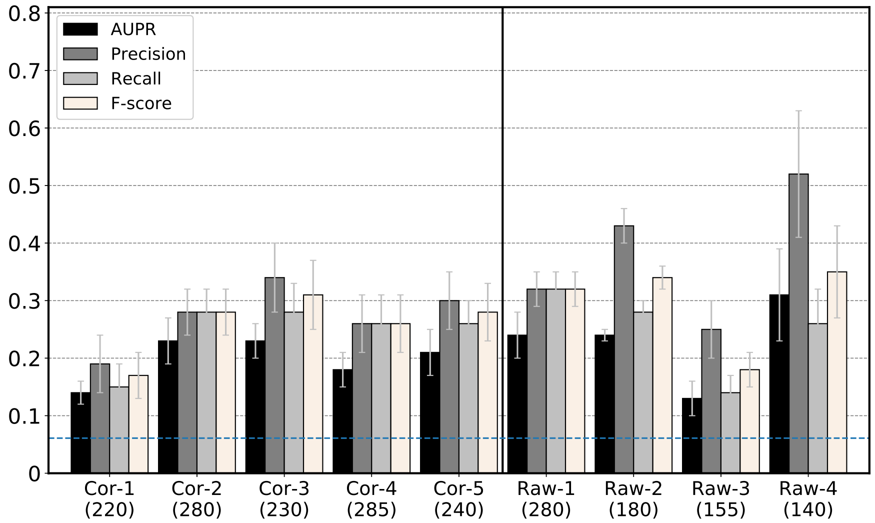

Supplementary Fig. S4: The prediction accuracy in terms of AUPR, precision, recall, and F-score of the nine predictive models for GTEx-BioGRID. The nine predictive models are named after their feature component. The number below the name of each predictive model represents the number of genes that are predicted as aging-related. The blue dashed line indicates the prediction accuracy scores expected by chance, i.e., the fraction of all genes in the ground truth data that are labeled as aging-related.

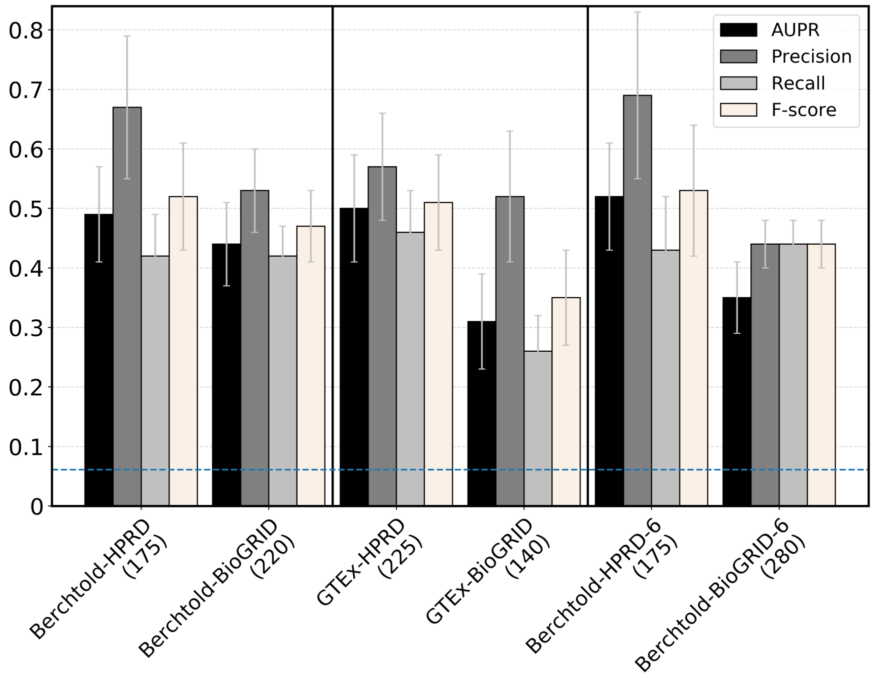

Supplementary Fig. S5: The prediction accuracy in terms of AUPR, precision, recall, and F-score of the four weighted dynamic aging-specific subnetworks plus Berchtold-HPRD-6 and Berchtold-BioGRID-6, each under its best predictive model. The number below each subnetwork name represents the number of genes that are predicted as aging-related by the corresponding subnetwork. The blue dashed line indicates the prediction accuracy scores expected by chance, i.e., the fraction of all genes in the ground truth data that are labeled as aging-related.

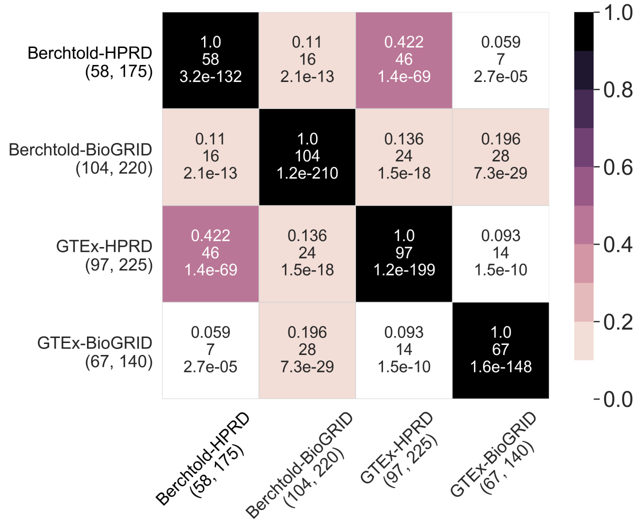

Supplementary Fig. S6: Pairwise overlap in terms of Jaccard indices of novel predictions for each pair of considered subnetworks. By novel predictions, we mean genes that are predicted as aging-related but are not present in GenAge. The two numbers in the parenthesis below each subnetwork name represent the number of novel predictions and the number of all predicted aging-related genes for the given subnetwork, respectively. In a cell, corresponding to a pair of subnetworks, the three numbers represent the Jaccard index (top), the raw number of genes in the overlap (middle), and the adjusted p-value indicating whether the overlap is statistically significantly high. The color shades are driven by Jaccard indices, where a darker color means a higher Jaccard index. Analogous results for true positives are shown in Fig. 3 in the main paper.

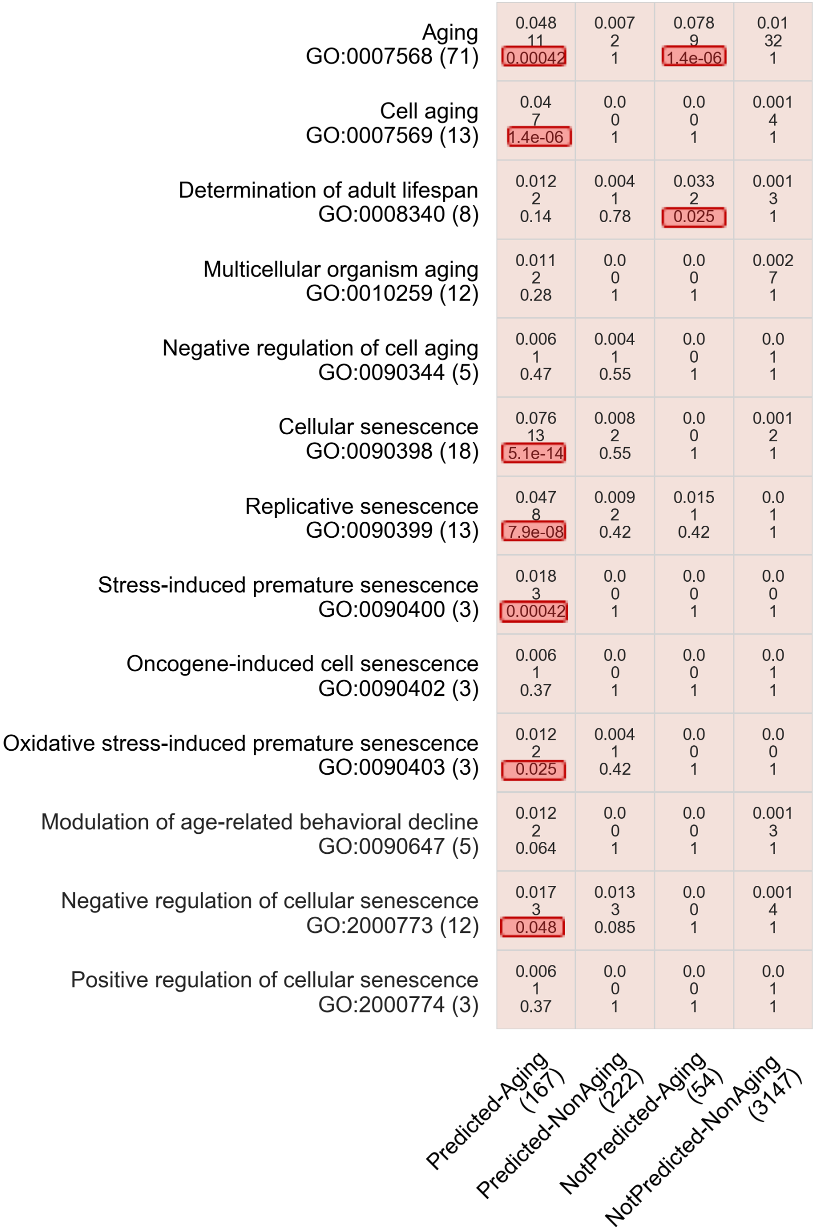

Supplementary Fig. S7: Enrichments of the four gene groups (x-axis) in the 13 aging-related GO terms (y-axis). The number below a GO term name or a gene group name represents the gene count in the pathway or gene group. In each cell, the three numbers represent the overlap size as measured by the Jaccard index (top), the raw number of genes in the overlap (middle), and the adjusted p-value indicating whether the overlap size is statistically significantly high, i.e., whether the given gene group is statistically significantly enriched in the given GO term. The adjusted p-values below 0.05 are highlighted in red. Note that there 4559 genes in the ground truth data. However, the total number of genes in these four gene groups is 3590, which does not equal to 4559. This is because for NotPredicted-Aging and NotPredicted-NonAging genes, we only consider those genes that are not predicted as aging-related by any of the four subnetworks. Then for these genes, we further consider whether they are labeled as aging- or non-aging-related in ground truth data. Analogous results for aging-related pathways are shown in Fig. 4 in the main paper.

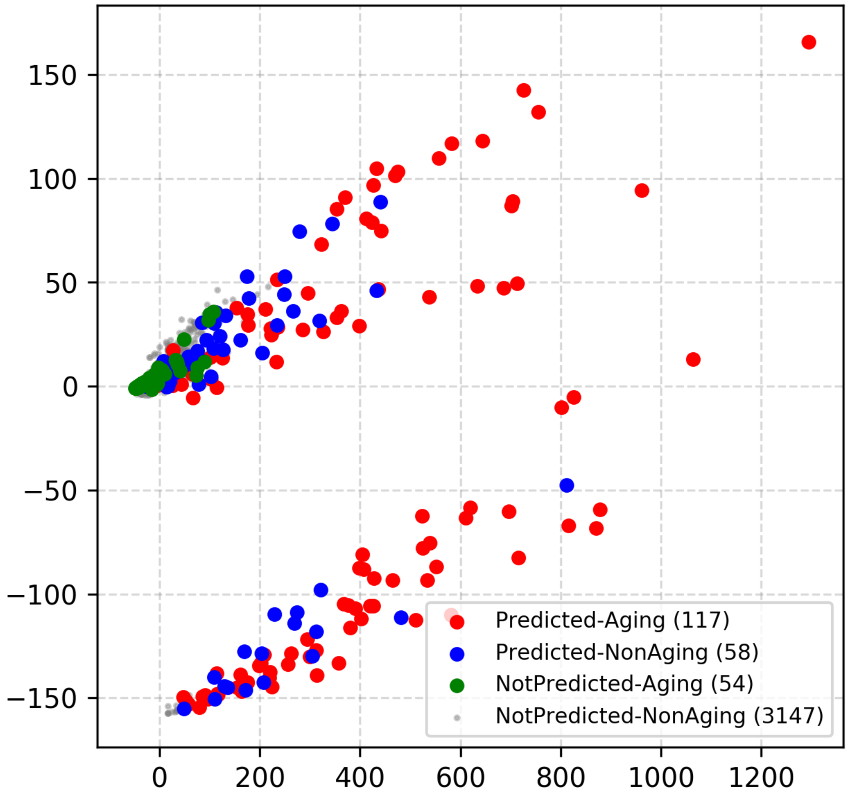

Supplementary Fig. S8: Illustration of topological similarities between the four gene groups (Predicted-Aging, Predicted-NonAging, NotPredicted-Aging, and NotPredicted-NonAging genes) for a given subnetwork by embedding their feature vectors into 2-dimensional (2D) vector space. The embedding presented in this figure corresponds to Berchtold-HPRD, similar embedding trends hold for the other three subnetworks (Supplementary Figs. S9, S10, and S11). The number in parenthesis behind a gene group name represent the number of genes in this gene group. For example, there are 117 genes are predicted as aging-related by Berchtold-HPRD that are also in GenAge. Note that in Fig. 4 and Supplementary Fig. S7, we have 167 "Predicted-Aging" genes, and Berchtold-HPRD contributes 117 such genes. Similarly, Berchtold-HPRD contributes 58 of the 222 "Predicted-NonAging" genes. For the two "NotPredicted" (i.e., "NotPredicted-Aging" and "NotPredicted-NonAging") gene groups, because they are not predicted by a subnetwork of interest, we take all genes in these two groups, i.e., 54 "NotPredicted-Aging" genes and 3147 "NotPredicted-NonAging" genes. Note that when mapping features into 2D space, we have tested tSNE and PCA and selected the visualization with the clearest pattern. This figure is generated using PCA.

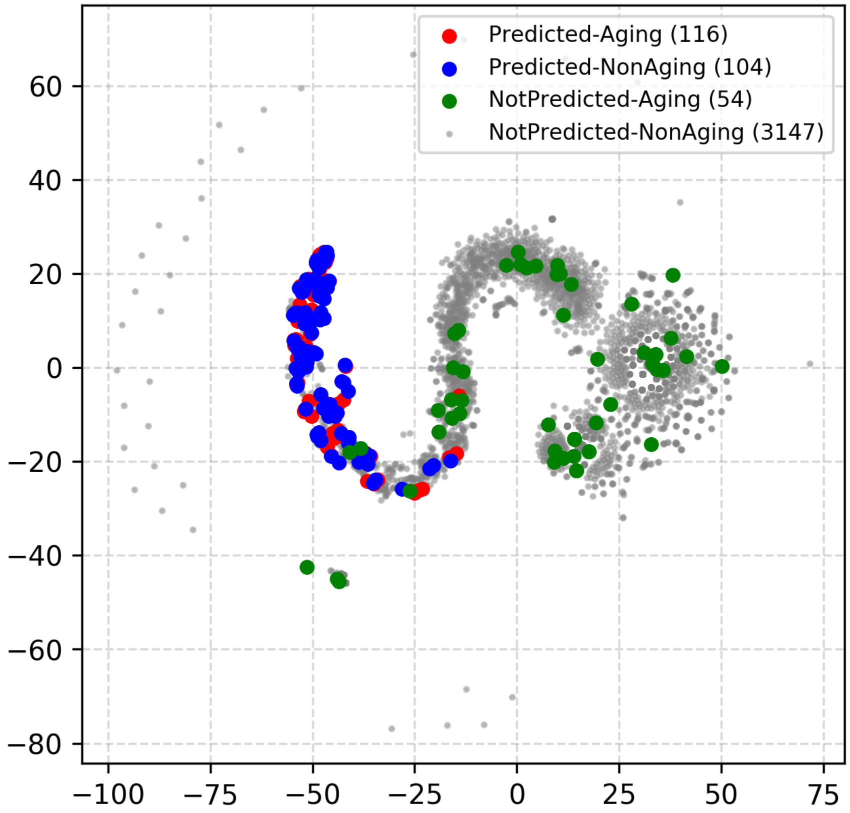

Supplementary Fig. S9: Illustration of topological similarities between the four gene groups (Predicted-Aging, Predicted-NonAging, NotPredicted-Aging, and NotPredicted-NonAging genes) for a given subnetwork by embedding their feature vectors into 2-dimensional (2D) vector space. The embedding presented in this figure corresponds to Berchtold-BioGRID, similar embedding trends hold for the other three subnetworks (Supplementary Figs. S8, S10, and S11). The number in parenthesis behind a gene group name represent the number of genes in this gene group. For example, there are 116 genes are predicted as aging-related by Berchtold-BioGRID that are also in GenAge. Note that in Fig. 4 and Supplementary Fig. S7, we have 167 "Predicted-Aging" genes, and Berchtold-BioGRID contributes 116 such genes. Similarly, Berchtold-BioGRID contributes 104 of the 222 "Predicted-NonAging" genes. For the two "NotPredicted" (i.e., "NotPredicted-Aging" and "NotPredicted-NonAging") gene groups, because they are not predicted by a subnetwork of interest, we take all genes in these two groups, i.e., 54 "NotPredicted-Aging" genes and 3147 "NotPredicted-NonAging" genes. Note that when mapping features into 2D space, we have tested tSNE and PCA and selected the visualization with the clearest pattern. This figure is generated using tSNE.

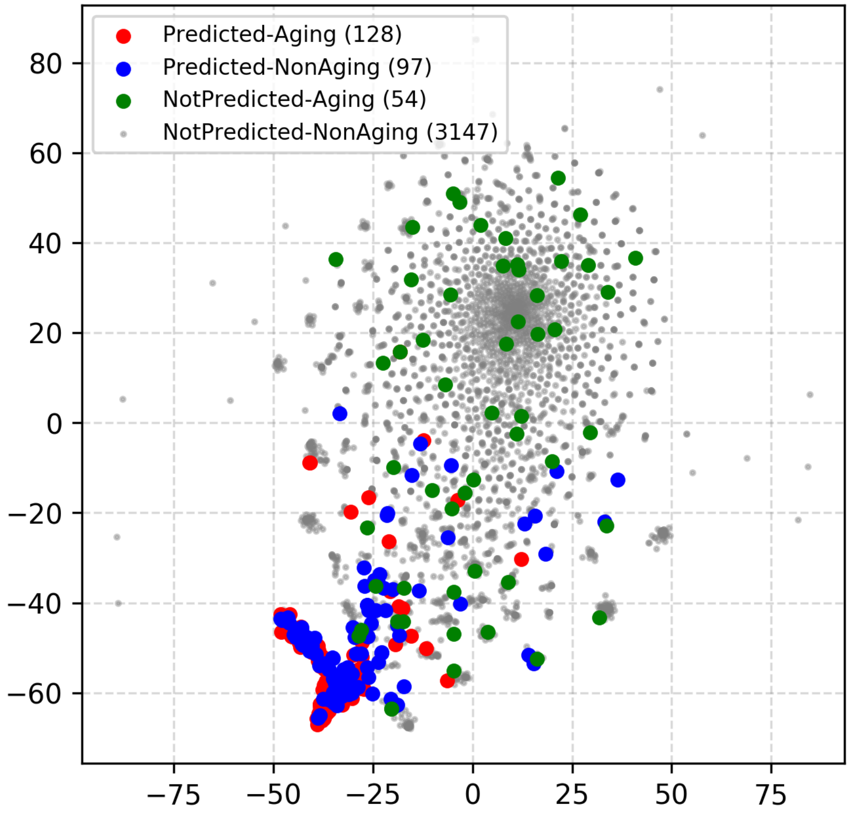

Supplementary Fig. S10: Illustration of topological similarities between the four gene groups (Predicted-Aging, Predicted-NonAging, NotPredicted-Aging, and NotPredicted-NonAging genes) for a given subnetwork by embedding their feature vectors into 2-dimensional (2D) vector space. The embedding presented in this figure corresponds to GTEx-HPRD, similar embedding trends hold for the other three subnetworks (Supplementary Figs. S8, S9, and S11). The number in parenthesis behind a gene group name represent the number of genes in this gene group. For example, there are 128 genes are predicted as aging-related by GTEx-HPRD that are also in GenAge. Note that in Fig. 4 and Supplementary Fig. S7, we have 167 "Predicted-Aging" genes, and GTEx-HPRD contributes 128 such genes. Similarly, GTEx-HPRD contributes 97 of the 222 "Predicted-NonAging" genes. For the two "NotPredicted" (i.e., "NotPredicted-Aging" and "NotPredicted-NonAging") gene groups, because they are not predicted by a subnetwork of interest, we take all genes in these two groups, i.e., 54 "NotPredicted-Aging" genes and 3147 "NotPredicted-NonAging" genes. Note that when mapping features into 2D space, we have tested *t*SNE and PCA and selected the visualization with the clearest pattern. This figure is generated using *t*SNE.

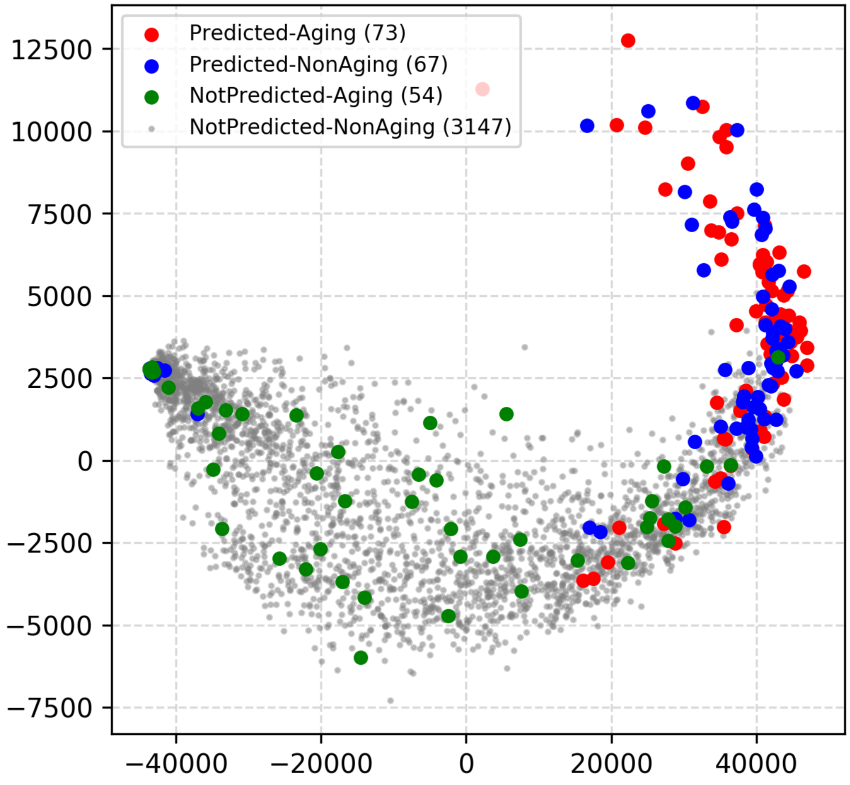

Supplementary Fig. S11: Illustration of topological similarities between the four gene groups (Predicted-Aging, Predicted-NonAging, NotPredicted-Aging, and NotPredicted-NonAging genes) for a given subnetwork by embedding their feature vectors into 2-dimensional (2D) vector space. The embedding presented in this figure corresponds to GTEx-BioGRID, similar embedding trends hold for the other three subnetworks (Supplementary Figs. S8, S9, and S10). The number in parenthesis behind a gene group name represent the number of genes in this gene group. For example, there are 73 genes are predicted as aging-related by GTEx-BioGRID that are also in GenAge. Note that in Fig. 4 and Supplementary Fig. S7, we have 167 "Predicted-Aging" genes, and GTEx-BioGRID contributes 73 such genes. Similarly, GTEx-BioGRID contributes 67 of the 222 "Predicted-NonAging". For the two "NotPredicted" (i.e., "NotPredicted-Aging" and "NotPredicted-NonAging") gene groups, because they are not predicted by a subnetwork of interest, we take all genes in these two groups, i.e., 54 "NotPredicted-Aging" genes and 3147 "NotPredicted-NonAging" genes. Note that when mapping features into 2D space, we have tested *t*SNE and PCA and selected the visualization with the clearest pattern. This figure is generated using PCA.

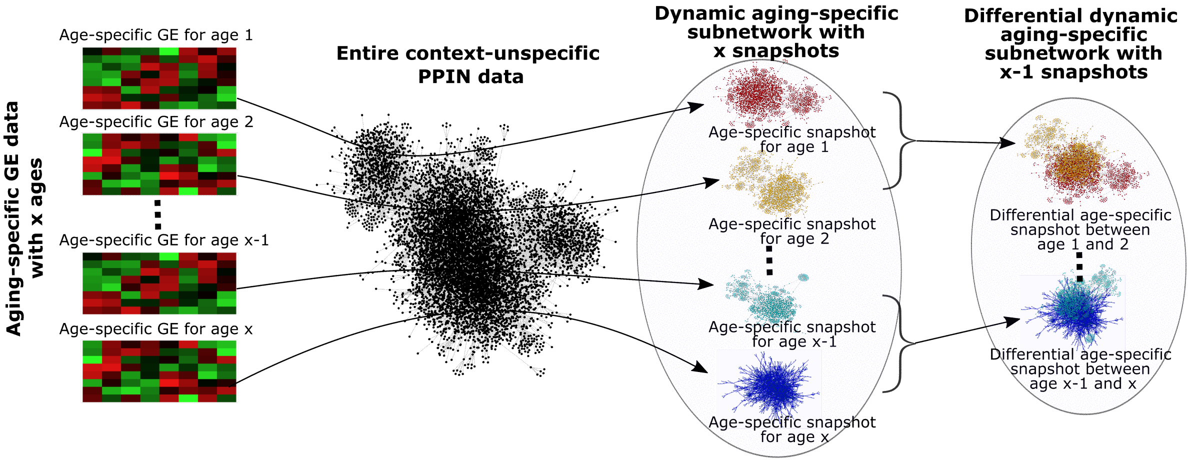

Supplementary Fig. S12: Workflow of how our weighted dynamic aging-specific subnetwork was inferred. See Section 5.1.3 in the main paper for detail.

References

M. Ashburner et al. Gene Ontology: tool for the unification of biology. *Nature Genetics*, 25(1):25–29, 2000.

Y. Benjamini and Y. Hochberg. Controlling the false discovery rate: a practical and powerful approach to multiple testing. *Journal of the Royal Statistical Society: series B (Methodological)*, 57(1):289–300, 1995.

J. Cao and S. Zhang. A bayesian extension of the hypergeometric test for functional enrichment analysis. *Biometrics*, 70(1):84–94, 2014.

Gene Ontology Consortium. The Gene Ontology resource: enriching a GOld mine. *Nucleic Acids Research*, 49(D1):D325–D334, 2021.

Q. Li and T. Milenković. Supervised prediction of aging-related genes from a context-specific protein interaction subnetwork. *IEEE International Conference on Bioinformatics and Biomedicine (BIBM)*, pp: 130–137, 2019.

Q. Li and T. Milenković. Supervised prediction of aging-related genes from a context-specific protein interaction subnetwork†. *IEEE/ACM Transactions on Computational Biology and Bioinformatics*, 2021.

Q. Li et al. Improved supervised prediction of aging-related genes via weighted dynamic network analysis. *BMC Bioinformatics*, 22(1):1–26, 2021.

L. v. d. Maaten and G. Hinton. Visualizing data using t-SNE. *Journal of Machine Learning Research*, 9 (Nov):2579–2605, 2008.

K. Newaz et al. Network analysis of synonymous codon usage. *Bioinformatics*, 36(19):4876–4884, 2020.

I. Rivals et al. Enrichment or depletion of a GO category within a class of genes: which test? *Bioinformatics*, 23(4):401–407, 2007.

F. Wilcoxon. Individual comparisons by ranking methods. *Breakthroughs in Statistics*, pages 196–202, 1992.

H.-F. Yu et al. Dual coordinate descent methods for logistic regression and maximum entropy models. *Machine Learning*, 85(1-2):41–75, 2011.

Q. Zheng and X.-J. Wang. GOEAST: a web-based software toolkit for Gene Ontology enrichment analysis. *Nucleic Acids Research*, 36(suppl_2):W358–W363, 2008.
